# Biological activity of a genetically modified BMP-2 variant with inhibitory activity

**DOI:** 10.1186/1746-160X-5-6

**Published:** 2009-02-02

**Authors:** Uwe Klammert, Joachim Nickel, Kristian Würzler, Christoph Klingelhöffer, Walter Sebald, Alexander C Kübler, Tobias Reuther

**Affiliations:** 1 Department of Cranio-Maxillo-Facial Surgery, University of Würzburg, Pleicherwall 2, 97070 Würzburg, Germany; 2 Department of Physiological Chemistry II, Biocenter, University of Wuerzburg, Am Hubland, 97074 Würzburg, Germany

## Abstract

**Background:**

Alterations of the binding epitopes of bone morphogenetic protein-2 (BMP-2) lead to a modified interaction with the ectodomains of BMP receptors. In the present study the biological effect of a BMP-2 double mutant with antagonistic activity was evaluated in vivo.

**Methods:**

Equine-derived collagenous carriers were loaded with recombinant human BMP-2 (rhBMP-2) in a well-known dose to provide an osteoinductive stimulus. The study was performed in a split animal design: carriers only coupled with rhBMP-2 (control) were implanted into prepared cavities of lower limb muscle of rats, specimens coupled with rhBMP-2 as well as BMP-2 double mutant were placed into the opposite limb in the same way. After 28 days the carriers were explanted, measured radiographically and characterized histologically.

**Results:**

As expected, the BMP-2 loaded implants showed a typical heterotopic bone formation. The specimens coupled with both proteins showed a significant decreased bone formation in a dose dependent manner.

**Conclusion:**

The antagonistic effect of a specific BMP-2 double mutant could be demonstrated in vivo. The dose dependent influence on heterotopic bone formation by preventing rhBMP-2 induced osteoinduction suggests a competitive receptor antagonism.

## Background

Heterotopic ossification is a pathological, non neoplastic process of bone formation at ectopic sites, especially inside mesenchymal soft tissues. The disorder can occur localized or generalized.

Local forms are mostly assigned to the entity of Myositis ossificans circumscripta and involve the skeletal muscles. As a result of trauma, often following total hip replacement, or due to neuropathic disorders, e.g. spinal cord lesions, an intramuscular osteogenesis occurs. The osteogenic stimulation of mesenchymal stem cells seems to be the cause, but the pathobiochemical pathways are not known exactly [1].

The generalized disorder Fibrodysplasia ossificans progressiva (FOP, syn. Myositis ossificans progressiva) is a rare connective tissue desease with autosomal dominant heredity. It is characterized by enchondral ossification of muscle, tendons and ligaments after simple injuries, e.g. intramuscular injection [2-4]. The influence of bone morphogenetic proteins on this disorder seems to be evident [5-8].

BMP-2 wild type binds to its cellular receptors via two distinct binding epitopes. The large epitope 1 is responsible for the high-affinity binding to the BMPR-IA receptor, the smaller epitope 2 provides the low-affinity binding to the receptor BMPR-II [9].

Different BMP-2 mutants with alterated binding epitopes were developed by Kirsch et al.. The in vitro evaluation of their biological activity, using ALP activity as a marker, revealed alterated effects for mutants of epitope 1 and epitope 2 as well. But only alterations of epitope 2 lead to a more or less strong inhibition of the activity of BMP-2 wild type. Necessary concentrations for half-maximal inhibition in the magnitude of BMP-2 wild type indicate a competitive antagonism at the same binding site [10].

In the present study a BMP-2 double mutant (A34D/D53A) was evaluated in vivo. This variant features alterations of amino acids at position 34 and 53: alanine was substituted by aspartate and aspartate by alanine, respectively. The mutation at position 34 mediates the inhibitoric activity via alterated interaction with BMPR-II, mutation at position 53 leads to a higher affinity to BMPR-IA than BMP-2 wild type. The consequence is a blockade of the BMP-2 receptor complex and thus a competitive antagonism with the wild type.

We are able to demonstrate that a BMP-2 double mutant provides an inhibitory activity opposite the BMP-2 wild type in a dose dependent manner. For this purpose a heterotopic implantation site (skeletal muscle) and BMP-2 wild type in a well known dose as an agonistic stimulus was chosen.

## Methods

### Origin of the proteins

The developement and expression of the utilized proteins in a bacterial expression system was performed by the department of physiologic chemistry II, University of Würzburg, as previously reported [11].

### Preparation of the protein-loaded implants

The collagenous carriers (extracted xenogous bone collagen) were prepared from equine cancellous bone using a procedure leant to the method described by Kuberasampath and Ridge [12]. The cylindric carriers with a diameter of 5 mm and a length of 10 mm were autoclaved, soaked with the protein solution and lyophilized.

### Animal studies

The presented in vivo study was performed using a heterotopic implantation site (lower limb muscle) of Sprague-Dawley rats in a split animal design. Control specimens (carriers coupled with 5 μg rhBMP-2) were implanted into prepared muscle cavities on the left side. Test specimens loaded with same dose rhBMP-2 (5 μg) as well as BMP-2 double mutant in increasing concentrations were placed at the same way into the opposite limb. Three groups with 6 individuals each were established, using doses of 10, 40 and 160 μg. Thus the number of animals was n = 18. After a period of 28 days the animals were sacrificed and the specimens were explanted.

### Examination of the implants

After explantation the mineralisation of the scaffolds was investigated radiographically in a 2-dimensional manner (Faxitron, 22 kV, 35 s). The radiograms were digitalized and the areas of new formed bone inside the specimens were measured and correlated to the well defined implant size. For this purpose the software Scion Image Alpha was used. The obtained data were compared and analysed statistically using a t-test for independent samples with p < 0,05.

Afterwards the specimens were processed histologically by decalcification, fixation, cutting and staining (Giemsa). The investigation was performed by optical microscopy and photography.

## Results

**Figure 1:**
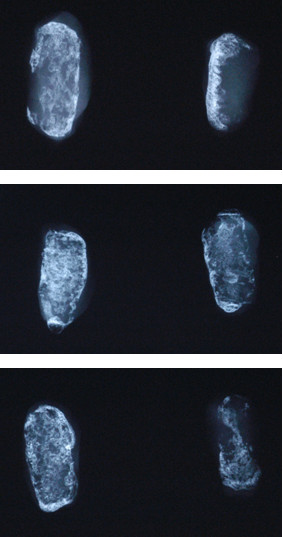
**Examples of X-rays of the specimens in pairs**. Left: control specimens with 5 μg rhBMP-2. Right: test specimens with 5 μg rhBMP-2 and 10 μg (top), 40 μg (middle), 160 μg (below) BMP-2 A34D/D53A.

The specimens were explanted with the surrounding soft tissue and X-rayed in pairs. The test specimens presented a slighter bone formation than the control specimens. The dimension of heterotopic bone formation was negative dependent on the dose of the BMP-2 double mutant A34D/D53A (Fig. 1).

**Figure 2:**
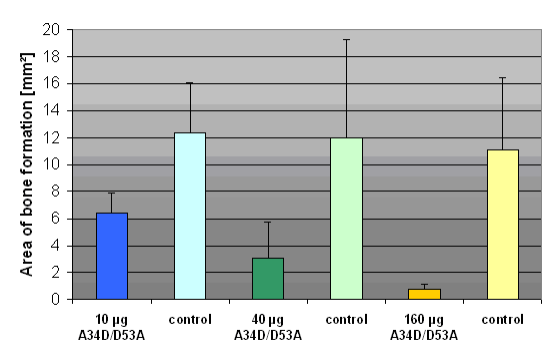
**Area of bone formation**. Illustration of the mean area (mm^2^) of newly formed heterothopic bone (error bar: 1 standard deviation).

**Figure 3:**
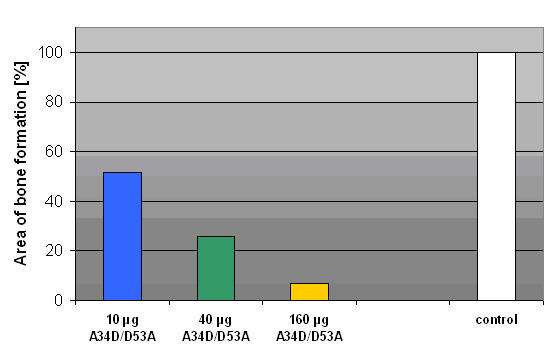
**Percentage of bone formation**. Percentage of newly formed heterothopic bone of the test specimens based on the control specimens which were set at 100%.

The areas of bone formation were portrayed 2-dimensionally after digitalisation of the X-rays. The data of the test specimens (5 μg rhBMP-2 and 10/40/160 μg BMP-2 double mutant A34D/D53A) were significant below the data of the control specimens (5 μg rhBMP-2). Further more a dose-dependent decrease of bone formation with increasing doses of A34D/D53A was detected: decrease of 48,2% (10 μg), 74,4% (40 μg) and finally 93,2% (160 μg). (Fig. 2, 3)

The test specimens as well as the controls displayed the cancellous structure of the carriers histologically. No foreign body reaction (e.g. giant cells) or other signs of inflammation were observed. Cartilaginous tissue as an indication of enchondral ossification was not detectable after the experimental course of 28 days.

**Figure 4:**
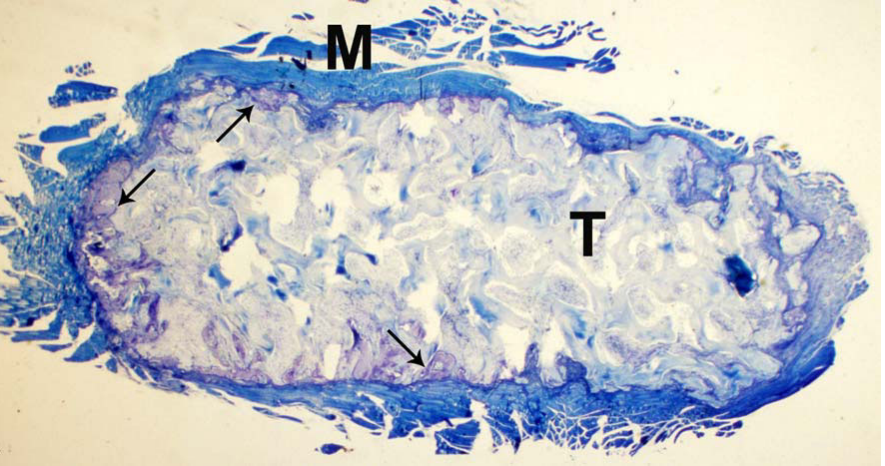
**Test specimen, histological section, Giemsa**. Carrier material (T) with surrounding skeleton muscle (M) of the implantation site, newly formed bone mostly at the marginal areas (arrows).

**Figure 5:**
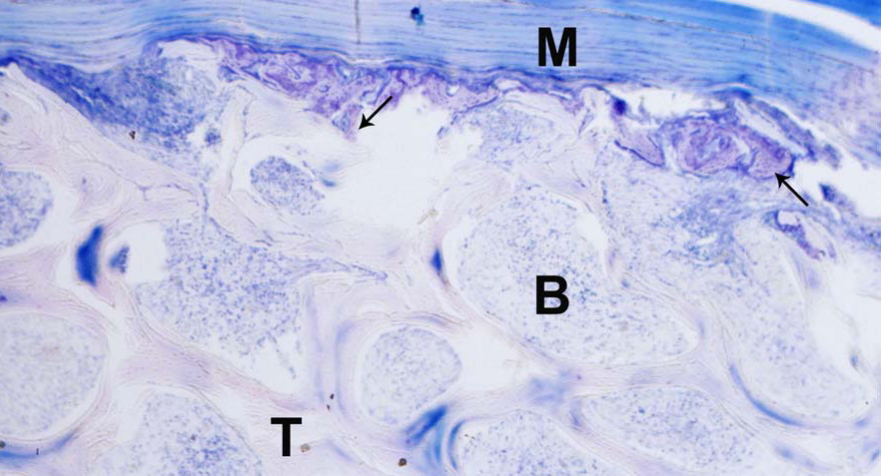
**Test specimen, histological section, Giemsa**. Carrier material (T) with surrounding skeleton muscle (M) of the implantation site, connective tissue (B) in the pores of the carrier, newly formed bone mostly at the marginal areas (arrows).

**Figure 6:**
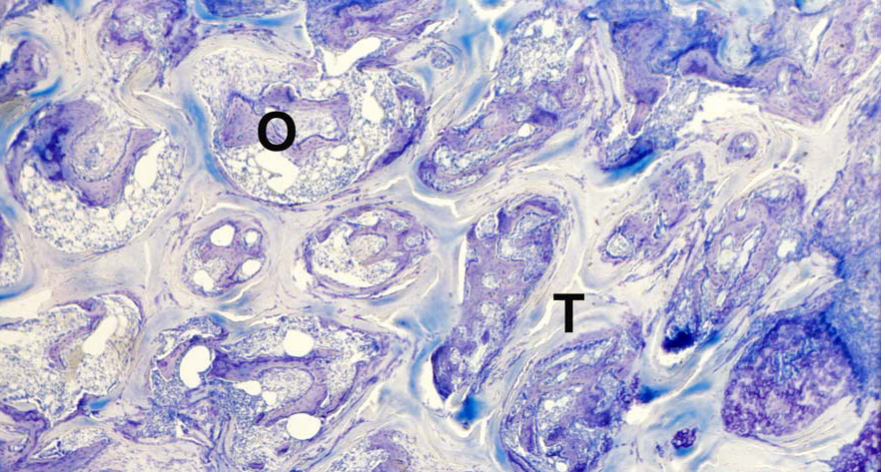
**Control specimen, histological section, Giemsa**. Carrier material (T) presenting much more mineralized matrix (O) within central areas of the scaffold.

**Figure 7:**
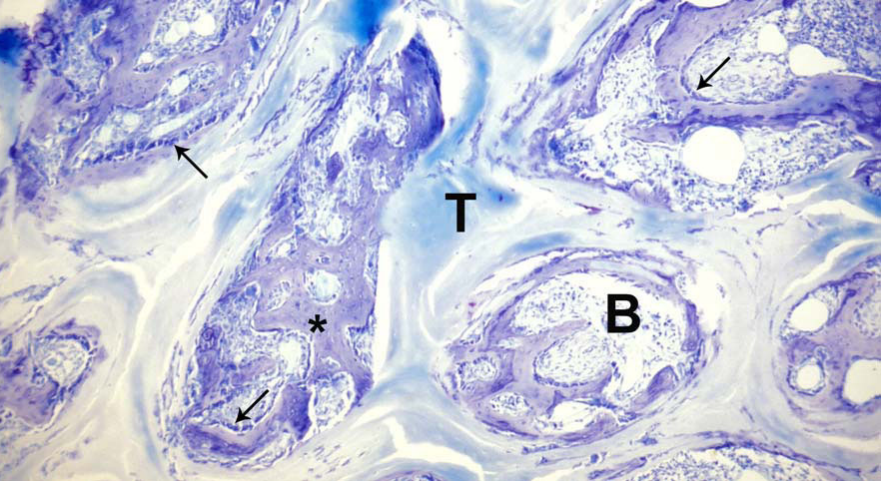
**Control specimen, histological section, Giemsa**. Carrier material (T) with connective tissue (B), presenting much more mineralized matrix (*) and osteoblastic cells (arrows) in central areas of the scaffold.

Furthermore the histological investigation revealed a slight bone formation mostly at the marginal areas of the test scaffolds. Most pores of the test scaffolds were filled with connective tissue. The control implants showed much more bone formation, not only at the margins but also within the central areas (Fig. 4, 5, 6, 7).

## Discussion

The effects of BMP-2 variants with antagonistic activity have already been described in vitro by using the promyeloblast cell line C2C12. A reduced activity of alkaline phosphatase after incubation with the BMP-2 double mutant A34D/D53A could be observed. The BMP-2 wild type was used as a receptor agonist to provide a simultaneous positive stimulus. Because the inhibitory variants work at concentrations similar to BMP-2, the competition for a common receptor binding site is most probably [10,11].

In the present study the inhibitory activity of the BMP-2 double mutant A34D/D53A could be demonstrated in vivo by inhibition of a specific osteoinductive stimulus (BMP-2 wild type) in a heterotopic implantation site. The area of newly formed bone by the principle of osteoinduction was significantly decreased in a dose-dependent correlation. Thus the previous in vitro results could be confirmed.

Several structurally distinct BMP inhibitors have been shown to modulate or block BMP activity within physiological conditions. Most of them are BMP binding proteins, e.g. Noggin, Chordin, Gremlin or Follistatin. Generally they regulate the activities and functions of different BMPs by forming complexes with them and thus they influence the binding of BMPs to their receptors. Some other BMP inhibitors work as receptor antagonists. These natural proteins – Inhibin and BMP-3 have been identified – bind to BMP receptors without activating the receptor complex [13].

Disorders of the BMP signal cascade and feedback control system seem to be involved in several musculoskeletal and extra-skeletal diseases. For example, an enhanced concentration of BMP-4 within the lesions of Fibrodysplasia ossificans progressiva was reported several times [3,5,6,14-16]. Further on there is evidence for BMP disorders concerning other deseases like osteoarthritis [17] or craniosynostosis [18-22].

The experimental arrest of heterotopic ossifications by application of BMP inhibitors has already been reported [23-26].

## Conclusion

The antagonistic effect of a specific BMP-2 double mutant could be demonstrated in vivo. The dose dependent influence on heterotopic bone formation by preventing rhBMP-2 induced osteoinduction suggests a competitive receptor antagonism. The development and clinical application of BMP antagonists like the current BMP-2 double mutant A34D/D53A could provide novel therapeutic options for treating BMP-associated disorders in the future.

## Competing interests

The authors declare that they have no competing interests.

## Authors' contributions

UK conceived the study, performed the surgery, evaluated the radiographical and hisological investigations, calculated the statistics and drafted the manuscript. JN and WS developed and prepared the proteins. KW conceived the study and helped to evaluate the radiographical and histological investigations. CK, ACK and TR participated in the study's design and coordination and helped to draft the manuscript. All authors read and approved the final manuscript.

## Ethics committee

The study was performed with the approval of the ethics committee and the local authority.
